# The tumor suppressor RASSF1A induces the YAP1 target gene *ANKRD1* that is epigenetically inactivated in human cancers and inhibits tumor growth

**DOI:** 10.18632/oncotarget.18177

**Published:** 2017-05-23

**Authors:** Adriana P. Jiménez, Annalena Traum, Thomas Boettger, Holger Hackstein, Antje M. Richter, Reinhard H. Dammann

**Affiliations:** ^1^ Institute for Genetics, Justus-Liebig University Giessen, D-35392 Giessen, Germany; ^2^ Department I-Cardiac Development and Remodeling, Max Planck Institute for Heart and Lung Research, D-61231 Bad Nauheim, Germany; ^3^ Clinical Immunology, Biomedizinisches Forschungszentrum Seltersberg, D-35392 Giessen, Germany; ^4^ German Center for Lung Research (DZL), Universities of Giessen and Marburg Lung Center, D-35392 Giessen, Germany

**Keywords:** RASSF1, YAP1, ANKRD1, tumor suppressor gene, epigenetic

## Abstract

The Hippo pathway regulates organ size, growth and comprises several tumor related factors, including the oncoprotein YAP1 and the tumor suppressor RASSF1A. *RASSF1A* is frequently epigenetically inactivated in cancer. In our study, we analyzed the effect of RASSF1A on the function of YAP1. Expression of YAP1 resulted in the downregulation of several tumor suppressor genes and induction of S-phase. Co-expression with RASSF1A normalized the expression levels of these tumor suppressors and induced a G0-G1 arrest and apoptosis. This effect was associated with the reduction of MDM2 and the increase of p53. These data suggest that the tumor suppressor RASSF1A inhibits the oncogenic potential of YAP1. Additionally, we could show that *ANKRD1* is a YAP1 target gene that is induced by RASSF1A. Further analysis revealed that *ANKRD1* is epigenetically inactivated in human cancer. ANKRD1 expression induced the expression of *TP53* as well as *BAX* and *CDKN1A* and reduced colony formation of cancer cells. We found that ANKRD1 interacts with p53 and is involved in the destabilization of MDM2. Additionally, our data indicate that the tumor-suppressive effect of ANKRD1 depends on the presence of p53. These results suggest that *ANKRD1* is a tumor-suppressive downstream target of the Hippo pathway that is epigenetically silenced in human cancer.

## INTRODUCTION

The Hippo pathway is a kinase cascade that regulates the organ size and plays an important role in cell differentiation, proliferation and cell death [1]. In this pathway, the tumor suppressor RASSF1A activates the mammalian STE20 like kinases 1 and 2 (MST1/MST2) via its Salvador/RASSF/Hippo domain [2, 3]. The hippo kinases MST1/MST2 in turn phosphorylate the large tumor suppressor kinases LATS1 and LATS2 [4]. Activated LATS1/LATS2 kinases phosphorylate the transcriptional regulator YAP1 in a HXRXXS context in S61, S109, S127, S164 and S381 [5–7]. Depending on the cellular context, YAP1 acts as a co-activator of transcription factors such as TEAD [8], SMAD [9] or TP73 [10] to regulate the expression of target genes that are involved in cell proliferation or apoptosis. YAP1 can be also phosphorylated by c-Abl on Y357 in response to DNA damage, thereby increasing the affinity of YAP1 to TP73 [11]*.* It was also reported that phosphorylated YAP1 interacts with 14-3-3 and is released into in the cytoplasm [12]. Phosphorylation of YAP1 has been correlated with its poly-ubiquitination and degradation [13]. It has been described that YAP1 *per se* rather acts as an oncogene and induces proliferation [8]. Furthermore, tumor tissues display an elevated YAP1 expression compared to normal tissues due to the amplification of the *YAP* gene locus [14, 15]. In lung cancer, YAP1 overexpression has been correlated with a poor prognosis [16]. YAP1 target genes, which promote its growth inducing function, are *CTGF* [8] or *cyclin D1* [17]. Previous reports have suggested that the tumor suppressive potential of YAP1 is due to its binding to TP73 [5, 10] and its regulation by RASSF1A leading to the expression of pro-apoptotic genes like *BBC3/PUMA* and *BAX* [5, 11]*.* The transcriptional regulator *Ankyrin repeat domain 1* (*ANKRD1*) has been characterized as a further YAP1 target gene [18, 19]. It has been demonstrated that Ankrd1 acts as a co-activator of p53 [20] and plays a role in apoptosis by inducing the expression of Bax in mice cardiomyocytes [21]. p53 regulates the expression of RASSF1A and MDM2 by positive and negative feedback loops [22–28]. Thus, the tumor-related functions of YAP1 may depend on different upstream activators and/or transcriptional co-factors resulting in the activation of distinct target genes [1, 5, 29, 30].

The scaffold protein RASSF1A is a versatile tumor suppressor that regulates microtubule stability, cell cycle progression and apoptosis [31]. RASSF1A induces apoptosis through the Hippo pathway by regulating the MST1/MST2 activation [3, 5, 32, 33]. According to several studies, *RASSF1A* is frequently epigenetically inactivated in several types of cancer including lung [34], skin cancer [35], prostate [36] and hepatocellular carcinoma [37]. Thus, *RASSF1A* silencing via its promoter hypermethylation may contribute to the oncogenic deregulation of YAP1.

To study the effect of RASSF1A on the transcriptional function of YAP1, we generated a YAP1 inducible cell system. Hereby, we demonstrated that RASSF1A co-regulates the expression of YAP1 target genes, including *ANKRD*1. Further analysis showed that ANKRD1 acts by destabilizing MDM2 and inducing p53 and BAX. Additional data suggest *ANKRD1* is epigenetically inactivated in cancer cells and its tumor suppressor role depends on p53.

## RESULTS

### YAP1 regulates the expression of tumor suppressor genes

**Figure 1:**
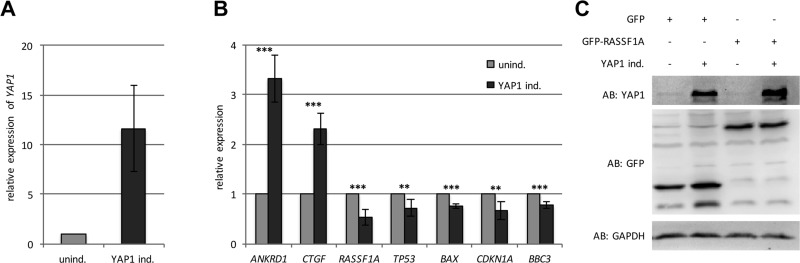
YAP1 regulates the expression of tumor-associated genes (**A**) Relative expression of *YAP1* in TREx293 clone pool after 24 h induction with 2 µg/ml Doxycyclin (YAP1 ind.) compared to uninduced cells (unind. = 1). All expression data were obtained by qRT-PCR and normalized to *GAPDH* level. (**B**) Relative expression of *ANKRD1, CTGF, RASSF1A, TP53, BAX, CDKN1A* and *BBC3* after 24 h induction of YAP1 (YAP1 ind.) compared to uninduced cells (unind.). (**C**) Western blot analysis of YAP1 in TREx293 cells after 72 h transfection with GFP-empty or GFP-RASSF1A with and without induction of YAP1. *p*-values *p < 0.05, **p < 0.01 and ***p < 0.001.

In order to investigate the effect of YAP1 on the expression of tumor suppressor genes, we generated an inducible Tet-On System in HEK293 cells (TREx293). These cells express low level of endogenous YAP1 and therefore we stably transfected *YAP1* (Figure 1). This system allows an induction of *YAP1* with doxycycline (Dox). Dox-treatment of the YAP1 inducible cells resulted in a 12-fold increase of the *YAP1* mRNA level compared to the control cells (Figure 1A) and the induction was confirmed on protein level (Figure 1C). Subsequently, we analyzed the expression of YAP1 target genes *(ANKRD1, CTGF, BAX, CDKN1A* and *BBC3)* by qRT-PCR. YAP1 significantly induced the expression of *ANKRD1* (3.3-fold) and *CTGF* (2.3-fold) (Figure 1B). Interestingly, a significant decrease in the expression of *BAX* (24%)*, CDKN1A* (33%) and *BBC3* (27%) was detected (Figure 1B). Moreover, YAP1 induction also resulted in significantly lower *RASSF1A* (48%) and *TP53* (29%) expression levels compared to untreated cells (Figure 1B). In contrast, the expression of YAP1 target genes was unaffected in Dox-treated TREx293 control cells (Supplementary Figure 1). Additionally, we also analyzed 12 individual YAP1 inducible TREx293 clones, which exhibited different *YAP1* levels upon Dox-treatment (Supplementary Figure 2) and analyzed the mRNA level of *ANKRD1, CTGF, BAX, CDKN1A* and *BBC3*. In these clones the *YAP1* level significantly correlated with *ANKRD1, CTGF, BAX* and *BBC3* expression (Supplementary Figure 3). For *CDKN1A,* a considerable trend toward significance was observed (Supplementary Figure 3). The suppressive effect of YAP1 was validated by luciferase promoter assays for *CDKN1A* (18%; *p* < 0.0001), *BAX* (7%; *p* = 0.02) and for a synthetic promoter with 13 conserved TP53 binding sites (28%; *p* < 0.001; Figure 3B).

**Figure 2:**
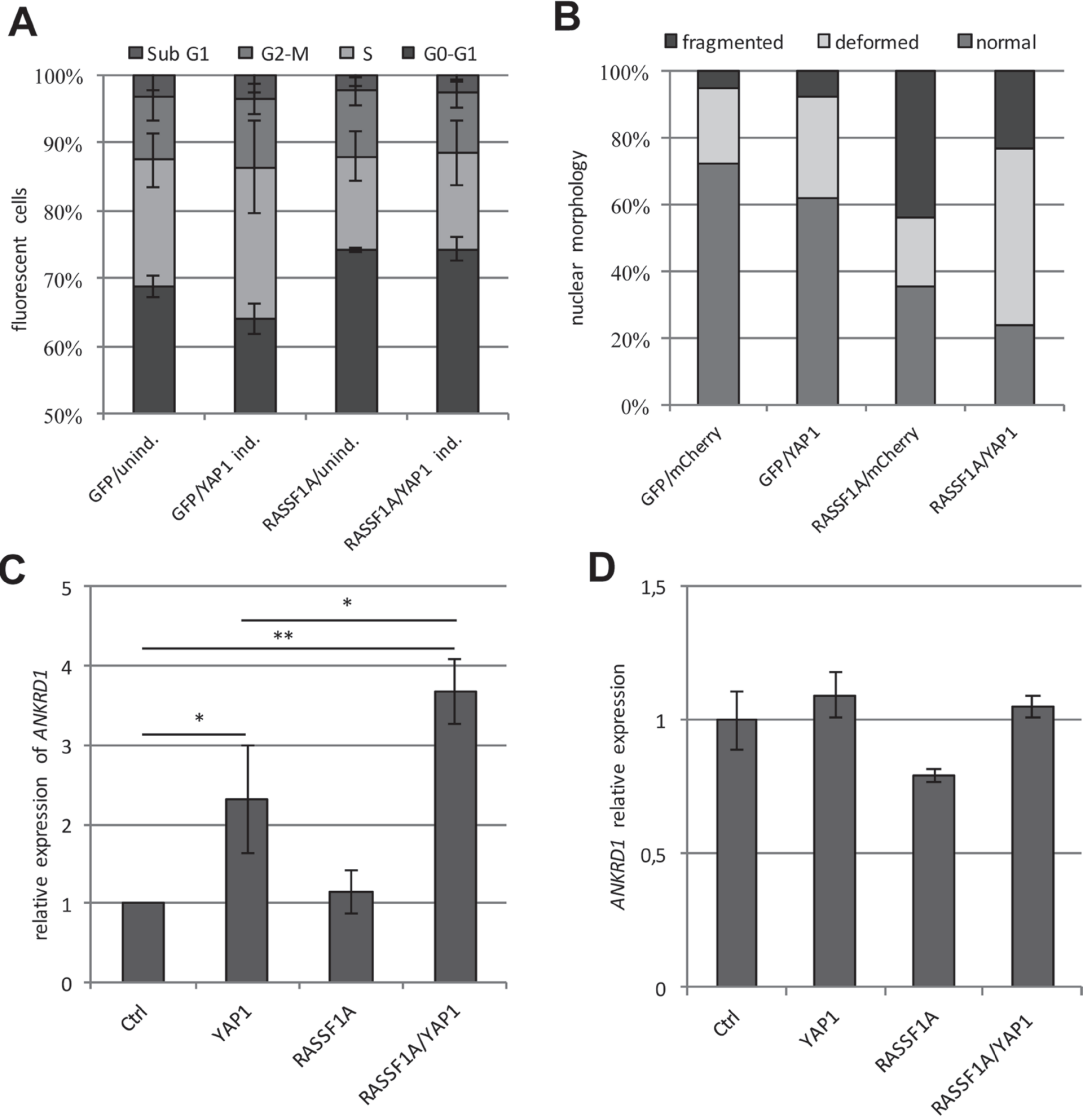
RASSF1A induces cell cycle arrest, apoptosis and *ANKRD1* expression (**A**) Flow cytometry analysis of YAP1 in TREx293 cells transfected with a GFP control or GFP-RASSF1A plasmid after 72 h treatment with Doxycycline (YAP1 ind.) or without (unind.). Cell cycle distribution was analyzed via flow cytometry using propidium iodid staining (*n* = 10^4^ cells). (**B**) Nuclear morphology after YAP1 and RASSF1A transfection. mCherry-YAP1 and GFP-RASSF1A and/or control plasmid were transfected in HEK293T cells; nuclei of transfected cells were analyzed by fluorescence microscopy after 72 h (*n* = 200 each). (**C**) Quantitative analysis of *ANKRD1* expression level in HEK293T cells transfected with control, *YAP1* and/or *RASSF1A* after 72 h. All expression data obtained by qRT-PCR were normalized to *GAPDH* and Ctrl was set 1. (**D**) Quantitative analysis of *ANKRD1* expression level in HeLa cells transfected with control, *YAP1* and/or *RASSF1A* after 72 h. *p*-values *p < 0.05, **p < 0.01 and ***p < 0.001 (*t*-test).

**Figure 3:**
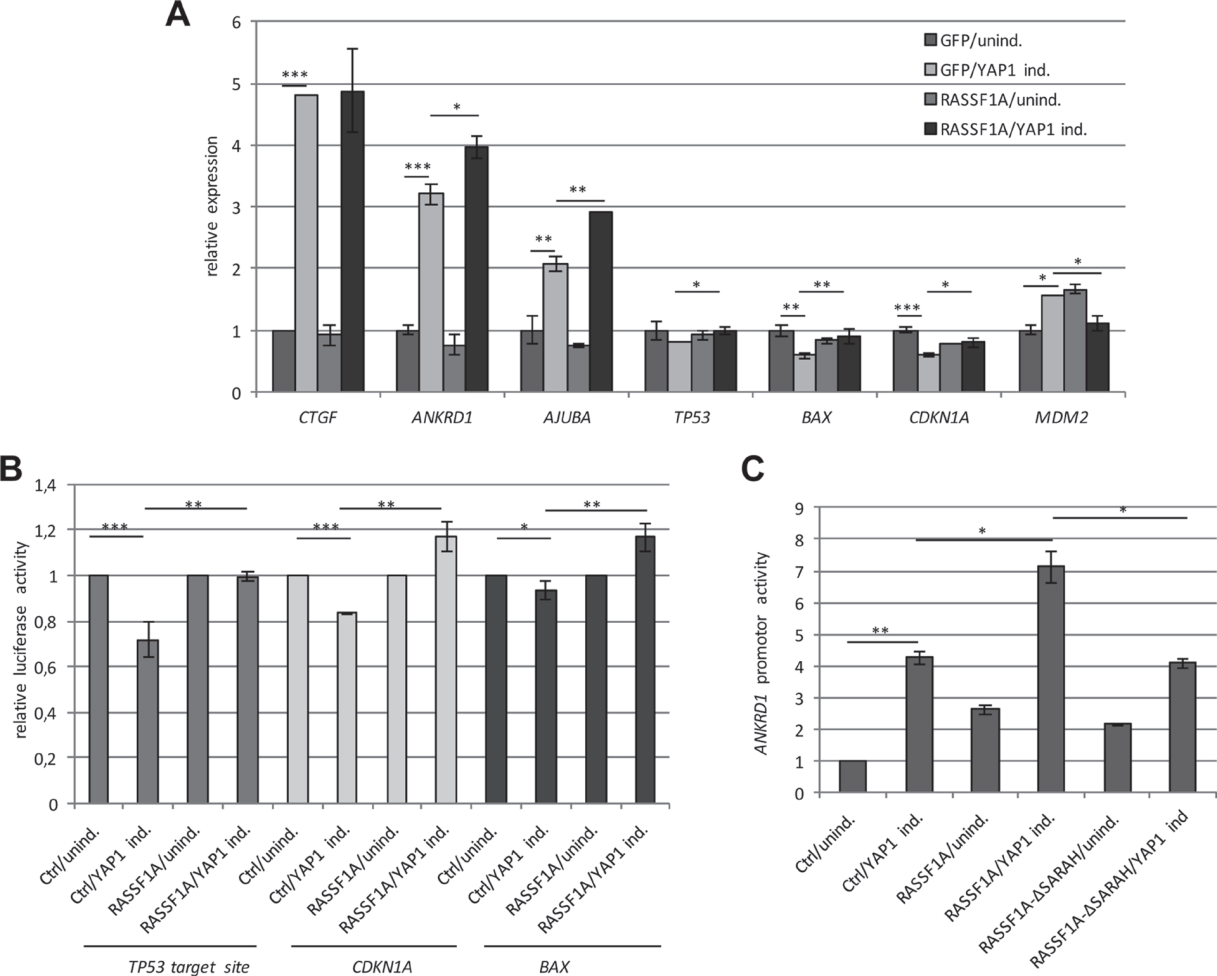
RASSF1A regulates the expression of YAP1 target genes (**A**) Expression of *CTGF, ANKRD1, AJUBA, TP53, BAX, CDKN1A* and *MDM2* was analyzed in TREx293 cells after 72 h transfection with GFP-empty or GFP-RASSF1A and without (unind.) or with YAP1 induction (YAP1 ind.). RNA levels in control cells (GFP/unind.) were set 1. (**B**) Firefly luciferase activity at the *BAX, CDKN1A* and synthetic p53 target site promoters with and without overexpression of RASSF1A and YAP1 induction (YAP1 ind.) or uninduced TREx293 cells (Ctrl/unind. = 1). All data were normalized to renilla luciferase activity. (**C**) Luciferase activity of *ANKRD1* promoter after RASSF1A, RASSF1A-ΔSARAH (deletion of SARAH domain) or control (Ctrl) transfection with and without induction of YAP1 in TREx293 cells. Promoter activity was normalized to firefly luciferase activity and control (Ctrl/unind = 1). *p*-values *p < 0.05, **p < 0.01 and ***p < 0.001.

### RASSF1A neutralizes the oncogenic potential of YAP1

The tumor suppressor RASSF1A plays an important role in the regulation of the Hippo pathway and is frequently hypermethylated in different human cancers [31]. We analyzed the methylation levels of core Hippo components such as *KIBRA, RASSF1A, MST1, MST2, WW45, LATS1, LATS2* and *YAP1* in five matched primary tumors (hepatocellular carcinoma) and normal liver samples by combined bisulfite restriction analysis (Supplementary Figure 4). *RASSF1A* was the only gene that exhibited a tumor-specific methylation pattern in these samples. *YAP1* was unmethylated in the tumor as well as the normal samples (Supplementary Figure 4). In the TREx293 cells, the promoter of *RASSF1A* is hypermethylated (Supplementary Figure 5). To analyze the effect of RASSF1A re-expression on YAP1 function, we transfected the YAP1 inducible TREx293 cells with GFP-RASSF1A or GFP (Figure 1C) and analyzed the cell cycle distribution via flow cytometry (Figure 2A). Doxycycline-dependent overexpression of YAP1 resulted in a significant reduction of the cell count in the G0/G1 phase and an increased number of cells in the S-phase (*p* < 0.001) compared to the untreated cells (Figure 2A). In contrast, the expression of RASSF1A counteracts the induction of the S-phase by YAP1 and arrests the TREx293 cells in G0/G1 phase (*p* < 0.001). Additionally via fluorescence microscopy, we observed that upon RASSF1A transfection, an increased number of apoptotic cells (fragmented and deformed nuclei) were detected (Figure 2B). Interestingly in HEK293T cells, the co-expression of RASSF1A and YAP1 significantly increased the expression of *ANKRD1* in comparison to the control and YAP1 by itself (*p* = 0.007 and *p* = 0.04 respectively; Figure 2C). In HeLa cells that express endogenous *RASSF1A* [34], *ANKRD1* induction by RASSF1A and YAP1 co-transfection was absent (Figure 2D). However for *CTGF* expression, RASSF1A co-expression had no additional effect compared to YAP1 alone (Figure 3A).

For the identification of novel RASSF1A-regulated YAP1 target genes, we performed a genome-wide expression analysis with the help of microarrays (Table 1). Hereby, we used RNA from 10^6^-sorted cells with GFP or GFP-RASSF1A and with or without YAP1 induction (Supplementary Figure 6). The Dox-dependent induction of YAP1 was confirmed on RNA and protein level (Supplementary Figure 6). We also verified that the YAP1 induction reduced the expression of *RASSF1A* by 50% (Figure 1B, Supplementary Figure 6D). Table 1 shows the expression levels of *ANKRD1, TP53, BAX* and *CDKN1A* by means of the microarray. Hereby, we confirmed the YAP1 and RASSF1A-induced upregulation of *ANKRD1* (3.4-fold). Furthermore, the downregulation of *TP53* (0.79-fold), *BAX* (0.63-fold) and *CDKN1A* (0.63-fold) through the induction of YAP1 were verified (Table 1). Interestingly, we observed that the re-expression of RASSF1A counteracted the YAP1 induced reduction of *TP53, BAX* and *CDNK1A* on RNA level in TREx293 cells (Table 1). These results were further validated by qRT-PCR (Figure 3A). Co-expression of RASSF1A and YAP1 significantly induced the expression of *ANKRD1*, *TP53*, *BAX* and *CDKN1A* compared to YAP1 only (Figure 3A). For *MDM2* mRNA level, RASSF1A and YAP1 co-expression significantly reduced the expression compared to YAP1 (Figure 3A). We further analyzed the effect of RASSF1A with the help of promoter assays (Figure 3B). RASSF1A significantly counteracted the YAP1 induced repression of synthetic *TP53* target, *CDKN1A* and *BAX* promoters (Figure 3B). With the microarray, we found that RASSF1A and YAP1 expression also normalized *MDM2* mRNA levels (Table 1). Subsequently, we analyzed protein levels of MDM2 by western blot analyses after YAP1 and/or RASSF1A overexpression in TREx293 cells and HEK293T cells (Supplementary Figure 6 and Figure 7). Interestingly in TREx293 cells, RASSF1A co-expression and YAP1 induction resulted in reduced MDM2 protein levels (30% reduction; Supplementary Figure 6E). This effect was confirmed by transient transfection of RASSF1A and YAP1 in HEK293T cells (Figure 7A–7B). Expression of RASSF1A and YAP1 resulted in a significant downregulation of MDM2 (40% reduction) and a 1.4-fold increase in BAX and p53 levels compared to control transfection (Figure 7A and 7B).

**Table 1:** RASSF1A and YAP1 regulated target genes

Gene	GFP/unind.	GFP/YAP1 ind.	RASSF1A/unind.	RASSF1A/YAP1 ind.	
*HIF1A-AS2*	1,00	7,70	1,16	10,90	**Top 10 up-regulated genes**
*ACTBL2*	1,00	2,72	1,16	4,85	
*COL12A1*	1,00	4,60	1,11	4,41	
*CTGF*	1,00	2,26	1,34	3,97	
*MT-TW*	1,00	2,90	2,58	3,64	
***ANKRD1***	**1,00**	**2,88**	**1,24**	**3,40**	
*CYR61*	1,00	2,66	1,43	3,26	
*CPA4*	1,00	2,74	1,24	3,22	
*AJUBA*	1,00	2,25	1,20	2,77	
*SPANXC*	1,00	1,69	1,55	2,67	
***CDKN1A***	**1,00**	**0,64**	**0,84**	**0,91**	
***TP53***	**1,00**	**0,79**	**0,95**	**0,95**	
***BAX***	**1,00**	**0,63**	**0,83**	**1,00**	
***MDM2***	**1,00**	**0,98**	**1,30**	**0,98**	
FOS	1,00	0,92	0,97	0,73	**Top 10 down-regulated genes**
*MAP2K6*	1,00	1,15	0,91	0,59	
*PINK1*	1,00	0,86	0,71	0,56	
*TP53I13*	1,00	0,81	0,84	0,51	
*GDF15*	1,00	0,60	0,72	0,46	
*FOXD3*	1,00	0,74	0,48	0,46	
*FGF21*	1,00	0,59	0,46	0,45	
*ANAPC1P1*	1,00	0,65	0,54	0,45	
*GH1*	1,00	0,57	0,65	0,41	
*MAP3K8*	1,00	0,40	0,89	0,39	

Expression of top 10 up- and downregulated genes and selected target genes (bold) after YAP1 induction (+Doxycyclin) and RASSF1A transfection in TREx293 cells. Levels were normalized to GFP/-Dox (unind. = 1) and microarray controls.

**Figure 4:**
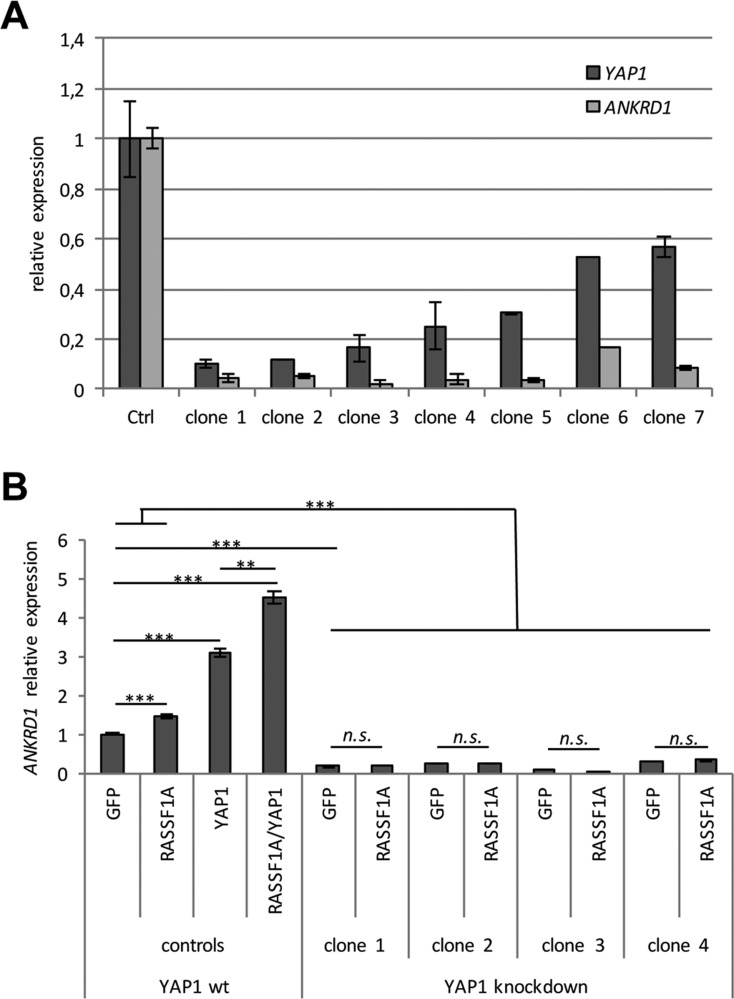
*YAP1* knockdown results in reduced *ANKRD1* levels (**A**) For the Crispr-Cas9 mediated *YAP1* knockdown, HEK293T cells were transiently transfected with 4 µg YAP1-pSpCas9(BB)-2A-Puro plasmid. After three weeks of 1 µg/ml puromycin selection, seven individual clones were expanded and RNA was isolated. *YAP1* and *ANKRD1* expression was analyzed by qRT-PCR and levels were normalized to *GAPDH* and a control clone (= 1). (**B**) Four clones with *YAP1* knockdown (> 85% reduction) were transfected with GFP and GFP-RASSF1A. As a control HEK293T cells were also transfected with YAP1 and/or RASSF1A. After 72 h RNA was isolated and *ANKRD*1 expression was analyzed by qRT-PCR. Levels were normalized to *GAPDH* and plotted relative to HEK293T GFP control (= 1). *p*-values for HEK293T cells: **p < 0.01 and ***p < 0.001.

**Figure 5:**
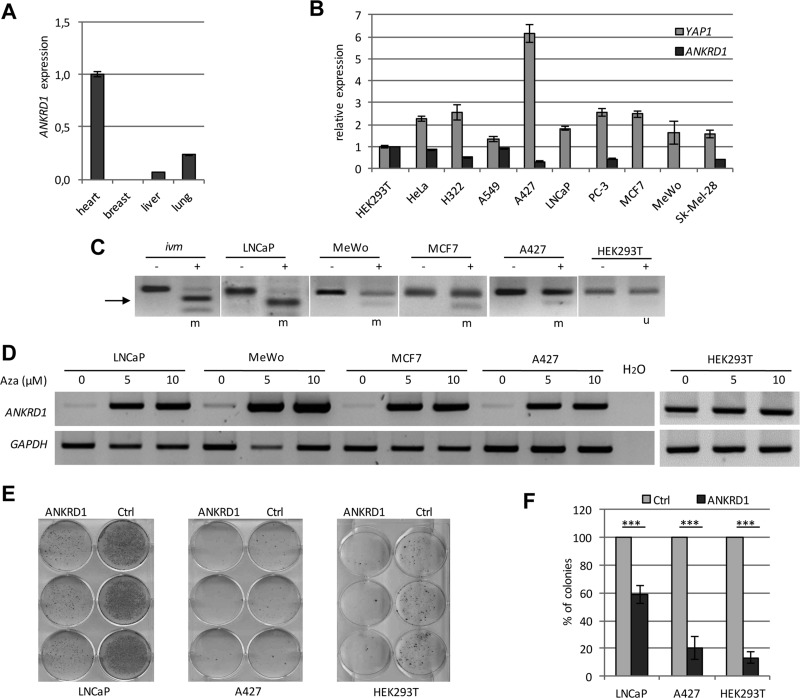
Epigenetic inactivation of *ANKRD1* in human cancer (**A**) Expression of *ANKRD1* was analyzed in normal heart, breast, liver and lung tissues by qRT-PCR. Expression was normalized to *GAPDH* and *ANKRD1* expression in heart was set 1. (**B**) Expression of *ANKRD1* and *YAP1* in cancer cell lines. *ANKRD1* and *YAP1* expression was analyzed in the indicated cell lines by qRT-PCR and normalized to *GAPDH* expression and levels in HEK293T were set 1. (**C**) DNA methylation analysis of the *ANKRD1* promoter region. The methylation was analyzed in LNCaP, MeWo, A427, MCF7 and HEK293T cells by combined bisulfite restriction analysis. Combined bisulfite restriction analysis covers one third of all CpGs in the analyzed region. PCR products were digested with *Taq*I (+) and mock digest (−). Length of PCR product: 139 bp and restriction products: 92 bp (arrow) and 47 bp. *ivm*: *in vitro* methylated DNA from HeLa cells; m: methylated u: unmethylated. (**D**) Re-expression of *ANKRD1*. The expression of *ANKRD1* and *GAPDH* in the indicated cancer cells was analyzed after 5 days of 5 Aza-2′-deoxycytidine treatment (0, 5 and 10 µM Aza) via semi-quantitative RT-PCR. (**E**) Ectopic expression of ANKRD1 reduced colony formation in cancer cells. Flag-empty vector (Ctrl) or Flag-ANKRD1/pCDNA4TO were transfected in LNCaP, A427 and HEK293T cells. After three weeks selection with G418 (LNCaP and A427) or Zeocin (HEK293T), colonies were stained and quantified. (**F**) Reduced colony count through ANKRD1. The colonies were counted and plotted relative to Ctrl (100%); ***p < 0.001.

**Figure 6:**
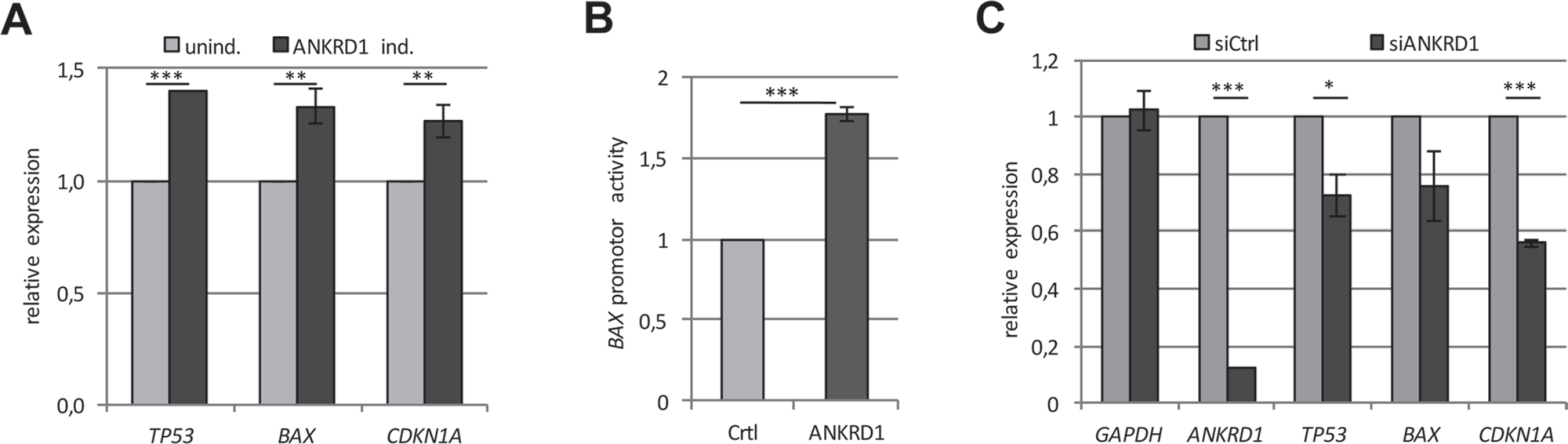
ANKRD1 regulates the expression of tumor suppressor genes (**A**) Relative expression of *TP53, BAX* and *CDKN1A* in ANKRD1 inducible TREx293 cells with and without ANKRD1 induction by Dox (2 µg/ml), normalized to *GAPDH* and uninduced TREx293 cells (unind. = 1). (**B**) ANKRD1 activates the *BAX* promoter. Relative luciferase activity of the *BAX* promoter after 24 h overexpression of Flag-empty vector (Ctrl = 1) or Flag-ANKRD1 was analyzed in HEK293T cells. (**C**) Relative expression of tumor suppressor genes after *ANKRD1* knockdown by RNAi. Expression of *ANKRD1*, *TP53, BAX* and *CDKN1A* in HEK293T cells treated with siRNA against *ANKRD1* (siANKRD1). RNA levels were analyzed by qRT-PCR and normalized to *GAPDH* and control siRNA (siCtrl = 1). *p*-values *p < 0.05, ** *p* < 0.01 and ***p < 0.001.

**Figure 7:**
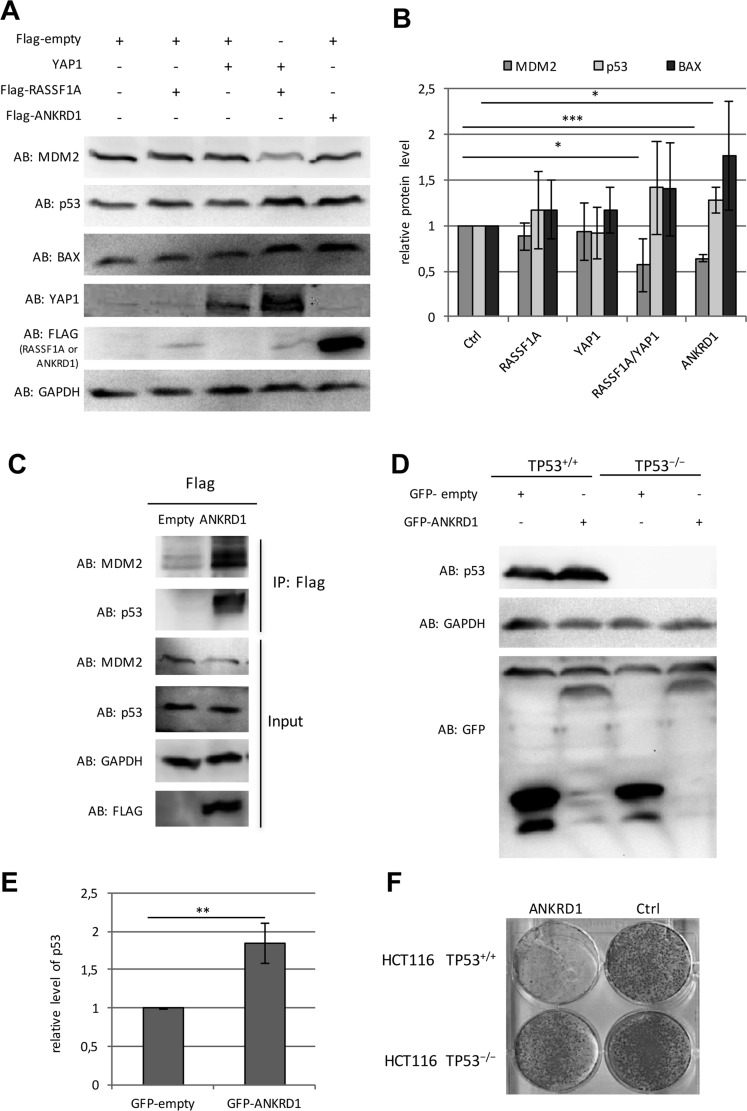
Tumor suppressive function of ANKRD1 (**A**) Representative Western blot of overexpression/co-transfection of Flag-empty, Flag-RASSF1A, Flag-YAP1, Flag-ANKRD1 after 72 h in HEK293T cells. (**B**) Quantification of protein levels of MDM2, p53 and BAX from five western blots of biological replicates with overexpression and co-transfection of Flag-empty (Ctrl), Flag-RASSF1A, Flag-YAP1, Flag-ANKRD1 in HEK293T cells after 72 h transfection. Data were quantified and normalized to GAPDH and control lysates via ImageJ (Ctrl = 1). (**C**) Co-immunoprecipitation of MDM2 and p53 with Flag-ANKRD1. Flag-empty and Flag-ANKRD1 were transfected in HEK293T cells. After 72 h protein lysates were extracted and analyzed by Western-blot (input) with indicated antibodies (AB). Flag-tagged ANKRD1 was precipitated with anti Flag beads and the co-precipitation of MDM2 and p53 was analyzed by western blot (IP: Flag). (**D**) Representative Western blot of overexpression/co-transfection of GFP-empty, GFP-ANKRD1 after 72 h in HCT116 *TP53*^*+/+*^ and HCT116 *TP53*^−*/*−^ cells. (**E**) Quantification of protein levels of p53 protein level from three western blots of biological replicates with co-transfection of GFP-empty (Ctrl) and GFP-ANKRD1 in HCT116 *TP53*^*+/+*^. Data were quantified and normalized to GAPDH and control lysates via ImageJ (Ctrl = 1). (**F**) Ectopic expression of ANKRD1 reduced colony formation in HCT116 TP53 wildtyp colon cancer cells. Flag-empty vector (Ctrl) or Flag-ANKRD1 were transfected in HCT116 *TP53*^*+/+*^ and HCT116 *TP53*^−*/*−^ cells. After two weeks of selection with G418, colonies were stained by Giemsa. *p*-values *p < 0.05, **p < 0.01 and ***p < 0.001.

The microarray analysis revealed additional genes that were upregulated by YAP1 and RASSF1A in TREx293 cells (Table 1). Some of the identified genes, like *HIF1A-AS2* and *AJUBA* are related to hypoxia. The Lim domain protein AJUBA has been linked to the Hippo pathway [38] and in our study we observed its induction by RASSF1A and YAP1 (Figure 3A)*.* Other genes such as *ACTBL2* and *COL12A1* are involved in the regulation of the cytoskeleton*. CYR61* and *CTGF* were previously identified as YAP1 target genes [8, 39]. Additionally, we found that RASSF1A and YAP1 induced the downregulation of *FOS, MAP2K6, GDF15, FOXD3, FGF21* and other genes (Table 1).

### *ANKRD1* is a tumor suppressive target gene of YAP1 and RASSF1A

Subsequently, we focused our experiments on ANKRD1, which is significantly upregulated through the combined expression of RASSF1A and YAP1 (Figures 2C and 3A and Table 1). The activation of the *ANKRD1* promoter following YAP1 and RASSF1A expression was confirmed by promoter assay (Figure 3C). However, when we utilized a RASSF1A construct that lacks the SARAH domain (ΔSARAH), the activation of the *ANKRD1* promoter was significantly reduced (Figure 3C).

Additionally, we performed a Crispr/Cas9 mediated knockdown of YAP1 in HEK293T cells and analyzed the expression levels of *YAP1* and *ANKRD1* in several clones (Figure 4). In seven clones, a considerable knockdown of *YAP1* compared to the control clone was detected (Figure 4A). Downregulation of *YAP1* significantly correlated with decreased *ANKRD1* mRNA levels (*p* = 0.05). Subsequently, we utilized four *YAP1* knockdown clones to analyze the effect of RASSF1A transfection on *ANKRD1* levels (Figure 4B and Supplementary Figure 7). In HEK293T control cells significantly increased *ANKRD1* levels were found after RASSF1A and/or YAP1 expression (Figure 4B). However, after *YAP1* knockdown the induced expression of *ANKRD1* was absent (Figure 4B). This indicates that RASSF1A regulates *ANKRD1* via YAP1 signaling.

Next, the expression of *ANKRD1* in normal tissues from heart, breast, liver and lung was analyzed (Figure 5). Although, in breast tissue *ANKRD1* was absent, a clear expression was found in heart, liver and lung tissues (Figure 5A). We also analyzed the expression of *ANKRD1* and *YAP1* in different human cancer cell lines, including lung cancer (H322, A549 and A427), prostate cancer (LNCaP and PC-3), breast cancer (MCF7) and skin cancer (MeWo and Sk-Mel-28). *YAP1* expression was observed in all analyzed samples (Figure 5B). However, *ANKRD1* expression was very low in several cancer cell lines (e.g. LNCaP, MCF7 and MeWo; Figure 5B and 5D). Thus we investigated the promoter methylation of *ANKRD1* in these cancer cells by combined bisulfite restriction analysis (Figure 5C). In HEK293T, in which *ANKRD1* is expressed, the analyzed CpG site was unmethylated (Figure 5C). In LNCaP, MeWo, A427 and MCF7 cells, the CpG site was methylated. We therefore analyzed the effect of a DNA methylation inhibitor (Aza: 5-Aza-2′-deoxycytidine) on the expression of *ANKRD1* in these cell lines (Figure 5D). Expression of *ANKRD1* was downregulated in LNCaP, MeWo, A427 and MCF7, but after treatment with 5 and 10 µM Aza, we observed a re-expression of *ANKRD1* (Figure 5D). These findings indicate that *ANKRD1* is epigenetically silenced in these cancer cells. In LNCaP cells that harbor a methylated *ANKRD1* promoter, the induction of *ANKRD1* by YAP1 was significantly reduced compared to HEK293T cells (Supplementary Figure 8).

Subsequently, we investigated the effect of ectopic *ANKRD1* expression on colony formation in LNCaP, A427 and HEK293T cells (Figure 5E). *ANKRD1* expression significantly reduced the number of colonies in LNCaP (42%; *p* < 0.001), in A427 (80%; *p* < 0.001) and in HEK293T (87%; *p* < 0.0001), compared to control transfected cells (Figure 5F). Consequently, we generated TREx293 cells with a doxycycline-dependent inducible *ANKRD1* expression. The induction of *ANKRD1* resulted in a significant upregulation of *TP53* (1.4-fold), *BAX* (1.3-fold) and *CDKN1A* (1.3-fold; Figure 6A). We also analyzed the effect of ANKRD1 expression on *TP53, BAX* and *CDKN1A* levels in HEK293T cells (Supplementary Figure 9A). We observed that expression of *ANKRD*1 caused a significant activation of *TP53* (1.5-fold), *BAX* (1.2-fold) and *CDKN1A* (1.2-fold) (Supplementary Figure 9A). A significant increased expression of *TP53* (1.2- to 1.9-fold) was also observed in LNCaP, MeWo, A549 and A427 after ANKRD1 overexpression (Supplementary Figure 9B). Moreover, we observed by promoter assays that ANKRD1 significantly activated the *BAX* promoter (Figure 6B). Next, we analyzed the effect of RNAi-mediated downregulation of ANKRD1 on the expression of *TP53, BAX* and *CDKN1A* (Figure 6C). We transfected HEK293T cells with siRNA against ANKRD1 and observed a reduction of 87% in *ANKRD1* mRNA levels compared to control siRNA (Figure 6C). Additionally, we observed that mRNA levels of *TP53* (28% reduction), *BAX* (24% reduction) and *CDKN1A* (44% reduction) were also downregulated after RNAi mediated-knock down of ANKRD1 (Figure 6C).

### The tumor suppressive function of ANKRD1 depends on p53

To further characterize the function of ANKRD1, we analyzed the protein levels of MDM2, p53 and BAX after ectopic ANKRD1 expression in HEK293T cells (Figure 7A and 7B). Here, we observed that ANKRD1 expression increased protein levels of p53 (1.3-fold) and BAX (1.8-fold). However, MDM2 was significantly downregulated (40% reduction) to similar levels as detected after *RASSF1A* and *YAP1* co-transfection (Figure 7A and 7B). Previously, it has been reported that ANKRD1 interacts with p53 [20]. We confirmed this interaction by co-immunoprecipitation of Flag-ANKRD1 with endogenous p53 (Figure 7C). Interestingly, we also found the co-immunoprecipitation of MDM2 with ANKRD1 (Figure 7C). Interaction of ANKRD1 with MDM2 and p53 was verified using GFP-tagged ANKRD1 (Supplementary Figure 10).

To further dissect the tumor suppressive function of ANKRD1 in the p53 pathway, we utilized the colon cancer cell line HCT116 that harbors a homozygote deletion of the *TP53* locus (HCT116 *TP53*^*−/−*^) and the corresponding HCT116 wild type (HCT116 *TP53*^*+/*+^) [40]. In HCT116 *TP53*^*−/−*^ cells no p53 protein could be detected (Figure 7D). We investigated the effect of ectopic ANKRD1 expression on p53 level and colony formation (Figure 7). In p53 wild type HCT116 cells, we observed a significant 1.8-fold induction of p53 by ANKRD1 (Figure 7D and 7E). Increase levels of p53 were also found after RASSF1A and YAP1 co-transfection (Supplementary Figure 11). Additionally, we revealed a reduction of MDM2 levels after overexpression of ANKRD1 or co-transfection of RASSF1A and YAP1 in HCT116, which confirmed our previous data in HEK293T (Supplementary Figure 11 and Figure 7). Co-transfection of RASSF1A and YAP1 also resulted in an induction of *ANKRD1* expression in HCT116 (Supplementary Figure 8). Subsequently, we analyzed the effect of ANKRD1 on colony formation in wild type and p53-depleted HCT116 cells (Figure 7F). In HCT116 *TP53*^*+/+*^ cells, ANKRD1 drastically reduced the colony formation compared to control transfected cells. In contrast, in HCT116 *TP53*^*−/−*^ cells this reduction in colonies was not found (Figure 7F). This data suggests that the tumor suppressive effect of ANKRD1 depends on the presence of p53.

## DISCUSSION

Cellular homeostasis is the precise balance between cell growth and death; disturbing this fine-tuning ultimately results in cancer. The Hippo pathway plays a crucial role in the regulation of organ size and carcinogenesis [41, 42]. The inactivation or activation of key regulators leads to an imbalance in this pathway, which may alter the pro-apoptotic effect of the Hippo pathway by switching it into the oncogenic function [43]. The downstream transcriptional activator of the Hippo pathway is YAP1, which is described as an oncogenic factor often activated in cancer [17, 44, 45]. In this study, we observed that the induction of YAP1 moderately reduced the expression of tumor suppressor genes including *TP53*, *BAX*, *CDKN1A* and *RASSF1A* (Figure 1 and Figure 3). Moreover, the overexpression of YAP1 elevates the mRNA level of the connective tissue growth factor *CTGF* and *MDM2* and induces S-phase (Figure 2 and Figure 3). The oncogenic function of YAP1 is mediated through the TEAD complex and the activation of proliferative genes like *CTGF* and *cyclin D1* [8, 17]. It has been reported that p53 directly regulates the expression of RASSF1A [26, 28] and that the loss of RASSF1A promotes the formation of an oncogenic YAP1-TEAD complex [46]. In this context, it is important to note that TREx293, which are derived from HEK293 cells exhibit a *RASSF1A* promoter hypermethylation (Supplementary Figure 5). The scaffold protein RASSF1A is an important tumor suppressive regulator of the Hippo kinases MST1/MST2 and the large tumor suppressor kinases LATS1/LATS2 [3, 5, 33]. RASSF1A interacts with MST1/MST2 through their common SARAH domain [2, 47]. *RASSF1A* is one of the most frequent epigenetically inactivated genes in different types of human cancer and acts as a prominent tumor suppressor [31, 34, 37]. We analyzed the promoter methylation of members of the Hippo pathway in hepatocellular cancer and observed tumor-specific hypermethylation of *RASSF1A*, although *YAP1* was unmethylated (Supplementary Figure 4). It has been shown that RASSF1A regulates YAP1 through the Hippo pathway [5, 48]. Here we revealed that re-expression of RASSF1A promotes its tumor suppressive function through the activation of pro-apoptotic and anti-proliferative YAP1 target genes (e.g. *BAX* and *CDNK1A*) in TREx293 cells (Figure 3). Matallanas *et al.* (2007) have shown that RASSF1A elicits apoptosis through the Hippo pathway by activating the transcription of pro-apoptotic genes via YAP1 and p73 [5]. We also observed that RASSF1A counteracts the oncogenic properties of YAP1 by preventing the YAP1-induced downregulation of *BAX, CDKN1A* and *TP53* (Figure 3 and Table 1). Moreover, RASSF1A induces apoptosis and accumulates the cells in G0/G1 phase (Figure 2). In A549 lung cancer cells, we observed the upregulation of *CDKN1A* and *BAX* after re-expression of RASSF1A confirming its apoptotic functions [49]. In this study interestingly, we found reduced protein levels of MDM2 after RASSF1A and YAP1 co-expression (Figure 7). However, mRNA levels of *MDM2* were not reduced compared to uninduced cells (Figure 3 and Table 1). MDM2 acts as E3 ubiquitin ligase that target p53 for proteasomal degradation [22]. It has been reported that RASSF1A negatively regulates MDM2 by disrupting the MDM2-DAXX-HAUSP complex and inducing the self-ubiquitination of MDM2 [50]. p53 regulates the transcription of *MDM2* by a positive feedback loop [23, 25]. We also observed that overexpression of the RASSF1A and YAP1 or their target gene ANKRD1 resulted in MDM2 degradation (Figure 7 and Supplementary Figure 11). Thus it will be interesting to analyze ANKRD1-induded degradation of MDM2 depends on p53.

YAP1, the main downstream regulator of the Hippo pathway, is highly expressed in cancer cells and involved in cell proliferation and tumorigenesis [16, 29, 51, 52]. Taking these findings into account, we observed that YAP1 induces the expression of *CTGF* (Figures 1 and 3). This growth inducing factor is a mitogen and was previously associated with the oncogenic properties of YAP1 [8]. Moreover, it is interesting to note that the co-expression of RASSF1A and YAP1 repressed the expression of several growth-associated genes including the transcription factors *FOS* and *FOXD3*, the mitogen-activated protein kinases *MAP2K6* and *MAP3K8* and the growth factors *FGF21, GH1* and *GDF15* (Table 1). It has been reported that K-Ras and YAP1 converge on the transcription regulator FOS and activate a transcriptional pathway, which is involved in the regulation of the epithelial-mesenchymal transition [53]. Additional targets of YAP1 signaling, including *CYR61* and *AJUBA* (Table 1 and Figure 3), have been described previously [38, 39, 54, 55]. Concerning AJUBA, it has been reported that it suppresses the proliferation of malignant mesothelioma via the Hippo pathway [56]. Interestingly, we observed that RASSF1A together with YAP1 induces the expression of *AJUBA* (Figure 3) and other YAP1 targets which have not been described so far (e.g. *HIF1A-antisense 2*, *ACTBL2*, *CPA4* and *COL12A1*; see Table 1). The precise tumor-related function of some YAP1 targets is still under investigation and may depend on expression levels, cell origin and/or the genetic and epigenetic background.

A YAP1 target gene that displays a tumor suppressive effect is *ANKRD1* (Figure 5 and Figure 7). In this study, we demonstrated that *ANKRD1* level is significantly increased after re-expression of RASSF1A in HEK293T and TREx293 that harbor a methylated *RASSF1A* promoter (Figure 2 and Supplementary Figure 6). However, in HeLa cells that express endogenous *RASSF1A* [34], RASSF1A and YAP1 overexpression had no effect on the expression level of *ANKRD1* (Figure 2). Consequently, we observed that after *YAP1* knockdown in HEK293T cells, the overexpression of RASSF1A did not induce the expression of *ANKRD1* (Figure 4). Knockdown of *YAP1* significantly correlated with the downregulation of *ANKRD*1 (Figure 4). Previously, the YAP1-dependent regulation of *ANKRD1* had been described [18, 57]. Since knockdown of *YAP1* abolished the additional induction of *ANRKD1* through RASSF1A, we concluded that RASSF1A regulates ANKRD1 through YAP1-signaling (Figure 4B). Moreover, the lack of the SARAH domain in RASSF1A exhibited a significant reduction in activation of the *ANKRD1* promoter (Figure 3). This indicates that the MST1/MST2 interaction domain of RASSF1A mediates the regulation of *ANKRD1* through the Hippo-pathway.

Interestingly, we found that *ANKRD1* is epigenetically inactivated in several cancer cells including lung and prostate cancer (Figure 5 and Supplementary Figure 8). We revealed that *ANKRD1* is expressed in heart, liver and lung tissue but is silenced in breast (Figure 5). Moreover, overexpression of *ANKRD1* moderately induced *TP53*, *BAX* and *CDKN1A* expression (Figure 6). Consequently, siRNA mediated knock-down of ANKRD1 resulted in the downregulation of these genes (Figure 6). To date, it has been reported that Ankrd1 works as a co-activator of p53 [20] and contributes to apoptosis by mitochondrial translocation of Bax and p53 phosphorylation [21]. In our study, we observed that ANKRD1 binds p53 and MDM2 and the expression of ANKRD1 reduced MDM2 protein levels leading to the stabilization of p53 and the increase of BAX (Figure 7). Our results suggest that ANKRD1 may act as a tumor suppressive target of the Hippo signaling pathway.

It has been suggested that ANKRD1 may function as a transcriptional co-regulator of *TP53* and reduces colony formation in hepatoma cells [20, 58]. Here we show that ectopic expression of ANKRD1 reduces colony formation in prostate, lung and HCT116 colon cancer cells (Figure 5F and 7F). Interestingly in HCT116 *TP53*^*−/−*^ cells that lack p53 protein, this reduction in colony formation was abolished (Figure 7F). This data indicates that the tumor suppressive effect of ANKRD1 depends on the presence of *p53*. Moreover, we show that ANKRD1 interacts with p53 and MDM2 (Figure 5 and Figure 7). Thus it will be interesting to dissect the ANKRD1/p53/MDM2 interaction and the induction of p53 by ANKRD1 in further detail.

In summary, we observed that RASSF1A promotes its tumor suppressive effect through activation of pro-apoptotic and anti-proliferative YAP1 target genes. Moreover, RASSF1A induces cell cycle arrest in G0/G1-phase and apoptosis. This suggests that the oncogenic potential of YAP1 may arise through the epigenetic silencing of *RASSF1A* and the deregulation of YAP1 in cancer. A RASSF1A and YAP1 target gene that displays tumor suppressive effects is *ANKRD1*. Since we observed epigenetic silencing of *ANKRD1,* this inactivation may contribute to the oncogenic effect of the deregulated YAP1-signaling in cancer. We also showed that ANKRD1 expression led to a downregulation of MDM2 and induced p53 levels. Moreover, our data show that the tumor suppressive function of ANKRD1 depends on the presence of p53.

## MATERIALS AND METHODS

### Cell lines and transfections and primary tissues

TREx293 cells, that stably express the Tet repressor (Thermo Fisher Scientific), were transfected with the expression vector pcDNA4TO-YAP1 and selected with Zeocin (Invitrogen). The cell lines TREx293, HEK293T, LNCaP, A427, HeLa and A549 were cultivated in DMEM or RPMI with 10% FCS at 37°C under 5% CO_2_ concentration. Colon cancer cell lines HCT116 p53^+/+^ and HCT116 p53^−/−^ were obtained from Thorsten Stiewe (University Marburg, Germany) and cultivated in DMEM [40]. The cells were transfected at a confluency of 60–80% in serum free media (GIBCO) with 4 or 10 µg DNA (3.5 cm, 10 cm plates respectively). HEK293T and TREx293 cells were transfected using PEI. LNCaP were transfected with Lipofectamin (Invitrogen). A549 were transfected using Turbofect (Thermo Fisher Scientific). A427 and HCT116 cells were transfected using X-tremGENE HP (Roche). HeLa were transfected using JetPEI (Polyplus) according to the manufacturer’s instructions. For the colony formation assay, the selection was performed using G418 (Biochrom) or Zeocin (Invitrogen) and colonies were stained with Giemsa (Fluka). Primary human hepatocellular carcinomas and matching normal tissue samples were obtained from patients of the University of Halle-Wittenberg and were previously described [59]. The local committee of medical ethics approved the use and all patients gave their consent.

### ANKRD1 knockdown

ANKRD1 knockdown was performed with small interfering RNA (Dharmacon). HEK293T cells were transiently transfected with either 50 pmol of a non-targeting siRNA control pool (*UGGUUUACAUGUCGACUAA; UGGUUUACAUGUUGUGUGA; UGGUUUACAUGUU UUCUGA; UGGUUUACAUGUUUUCCUA*) or with 50 pmol of the siRNA for ANKRD1 (*CUACAAGACCUCUCGC AUA; GAACCAAAGCAAUAUUCGA; CGAAUUCCGUGA UAUGCUU; GCUAUAAGAUGAUCCGACU*) using the Lipofectamine^®^ RNAiMAX (Invitrogen). The cells were harvested after 4d for RNA isolation and expression analysis.

### *YAP1* knockdown by Crispr-Cas9

Two guide oligos for *YAP1* genomic knockout were generated using the protocol by Ran *et al.* and the online tool *http://tools.genome-engineering.org*. Sequences for guide oligo 1: upper CACCGCATCAGATCGTGCACGTCCG and lower AAACCGGACGTGCACGATCTGATGC and guide oligo 2 upper CACCGCAGCAGCCGCCGCCTCAAC and lower AAACGTTGAGGCGGCGGCTGCTGC. YAP1 guide oligos were cloned into pSpCas9(BB)-2A-Puro via Bbs1 sites. Expression of Cas9 was verified by western blotting through its Flag-Tag.

For the Crispr-Cas9 mediated *YAP1* knockdown, HEK293T cells were transiently transfected with 4 µg YAP1-pSpCas9(BB)-2A-Puro plasmid. The transfected cells were further selected with Puromycin (PAA laboratories) in a final concentration of 1 µg/ml. After three weeks, individual clones were isolated and expanded. The clones with a reduced *YAP1* expression (85% reduction) were transfected using PEI with GFP and GFP-RASSF1A for further characterization by qRT-PCR.

### Constructs

Indicated full length cDNAs and promoters were used in the representative vectors: pEGFP-C2; pEGFP-RASSF1A; pEGFP-ANKRD1; pCMV-Flag; pCMV-Flag-RASSF1A; pCMV-Flag-YAP1; pCMV-Flag-ANKRD1; pcDNA4TO; pcDNA4TO-YAP1; pcDNA4TO-ANKRD1; pRL-Null; pRL-ANKRD1; pGL2-basic; pGL-p21; pGL-BAX; pGL-MDM2; pGL-p53. The correctness of all constructs was confirmed through conventional sequencing.

### RNA expression analysis

RNA was isolated with Isol-RNA Lysis Reagent (5 Prime). 2 µg of RNA was digested with DNase I (Thermo Fisher Scientific) and reversely transcribed to cDNA with the M-MLV Reverse Transcriptase (Promega) for further analysis. The primers are listed in Supplementary Table 1. The qPCR data were obtained with SYBR- Select (Thermo Fisher Scientific) and measured in triplicates in Rotor Gene from Corbett Research. The samples were normalized to *GAPDH* and are displayed as ratio of the respective control sample.

### Generation of YAP1 and ANKRD1 stable cell lines

We created the stable cell lines in the TREx293 Tet-On inducible system (Clontech). cDNA of *YAP1* or *ANKRD1* were cloned into pcDNA4TO Myc vector and transfected into the TREx293 cells. The cells were cultivated in DMEM with 10% tetracycline free serum (Biochrom) and 1% penicillin and streptomycin (GIBCO) under the same conditions as described above. The selection of the clones was performed using blasticidin (5 µg/ml, Roth) and zeocin (500 µg/ml, Invitrogen). The induction of *YAP1* or *ANKRD1* was performed using doxycycline (2 µg/ml, Invitrogen).

### Methylation analysis

The methylation of *ANKRD1, RASSF1A, MST1, MST2, WW45, LATS1, LATS2, YAP1 and KIBRA* promoters were analyzed via combined bisulfite restriction analysis (CoBRA) as described previously [34, 60]. Bisulfite specific primers are listed in Supplementary Table 1. 2 µg DNA were bisulfite treated to convert unmethylated cytosine into uracil. The region of interest from DNA of each cell line was amplified by PCR using the specific primers for bisulfite DNA (Supplementary Table 1). The obtained PCR products were further digested with *Taq*I (Thermo Fisher Scientific) for 1 h at 65°C. The digested products were resolved on a 2% agarose gel.

### Western blot, antibodies and immuno-precipitation

The cell lysates were loaded on 10% SDS gels and blotted onto a PVDF membrane (Amersham). Santa Cruz Antibodies: anti YAP1 H-125 (1:200), anti GAPDH FL-335 (1:2000), anti p53 Do-I (1:2000) and anti ANKRD1 H-120 (1:200). Anti mp53 1C12 (1:1000) from Cell Signaling, anti MDM2 Ab-2 (1:1000) from Calbiochem. Anti BAX 06-499 (1:1000) from Millipore and anti GFP rabbit polyclonal serum (1:2000). The protein levels were quantified using ImageJ after subtraction of the background and normalized to GAPDH levels and control lysates.

For the immunoprecipitation, HEK293T cells were transiently transfected either with 10 µg of pCMV-Flag-ANKRD1 or pEYFP-ANKRD1 and empty vector control, respectively. The cell lysates were incubated overnight with anti-flag M2 agarose beads (Sigma) or with anti-GFP agarose beads (Chromotek). The beads were washed twice with cold TBS before denaturation. The cell lysates (input) and IPs were separate by SDS-PAGE. The interactions were further analyzed by western blotting using the specific antibodies.

### FACS and microarrays

Cell cycle distribution was analyzed via flow cytometry (BD FACSCanto) using propidium iodid (Sigma) and pEGFP or pEGFP-RASSF1A transfected TREx293 cells (−/+ doxycycline). For the microarrays, 10^6^ cells were sorted in BD Biosciences FACS Aria III flow cytometer. The purity (GFP signal) of the sorted cells was analyzed after sorting as shown in Supplementary Figure 6. RNA from the sorted cells was obtained using Isol-RNA Lysis Reagent (5 Prime); 250 ng RNA was employed for the expression analysis by Affymetrix arrays: HuGene version 2.0 (Affymetrix). Microarray data are available in the ArrayExpress database (www.ebi.ac.uk/arrayexpress) under accession number E-MTAB-4781.

### Promoter assays

The *ANKRD1* promoter (600 bp) was cloned into pRL-null vector. The promoters of a synthetic TP53 target site (250 bp with 13 p53 binding sites: GGACTTGCCT), *p21* and *BAX* cloned into pGL-vectors were obtained from Lienhard Schmitz (Justus-Liebig-University, Germany). The transfection efficiency was controlled using the corresponding empty control vector (300 ng firely or renilla luciferase controls, respectively). The measurements were performed using the Dual-Luciferase Reporter Assay System (Promega) and the microplatte illuminometer ORION L (Berthold). The obtained data were normalized to the corresponding control vectors.

### Fluorescence microscopy

HEK293T cells were seeded onto cover slips in 6-well plates and transfected with pEGFP or pEGFP-RASSF1A together with mCherry or mCherry-YAP1. After 72 h, cells were washed twice with PBS (Sigma) and fixed by 3.7% formaldehyde (Roth). After washing with PBS, the cells were permeabilized with 0.2% TritonX (Roth). DAPI (1 µg/ml, Serva Electrophoresis) was used for nuclear staining. The nucleus morphology of *n* = 200 cells were analyzed using the 63× zoom lens of Axio Observer.Z1 microscope (Zeiss) with a camara Orca-ER (Hamamatsu) and Volocity software.

### Statistical analysis

Statistical and correlations analyses were performed using R version 3.1.3 (R Foundation). The data are presented as the means of biological triplicates ± S.D. The *p*-values were quantified by Student’s unpaired *t*-test or by *Chi* square test for the FACS data. The differences are considered significant if: *p < 0.05; **p < 0.01; ***p < 0.001.

## SUPPLEMENTARY MATERIALS FIGURES AND TABLE



## Abbreviations

YAP1: (Yes-Associated Protein 1)
ANKRD1: (Ankyrin Repeat Domain 1/CARP)
RASSF1A: (Ras Association Domain Family 1A)
TP53: (Tumor Suppressor p53)
CDKN1A: (Cyclin-dependent Kinase Inhibitor 1A/p21)
BAX: (BCL2-associated X protein)
MDM2: (human homologue of mouse double minute 2)
BBC3: (BCL2 binding component 3/PUMA)
