# Supplementary material for: The tumor suppressor RASSF1A induces the YAP1 target gene ANKRD1 that is epigenetically inactivated in human cancers and inhibits tumor growth
Source: Oncotarget. 2017 May 23;8(51):88437–52. doi: 10.18632/oncotarget.18177 (PMC5687617; doi:10.18632/oncotarget.18177)
Supplement: Supplementary file 1 [file oncotarget-08-88437-s001.pdf]

The tumor suppressor RASSF1A induces the YAP1 target gene *ANKRD1* that is epigenetically inactivated in human cancers and inhibits tumor growth

Supplementary Materials

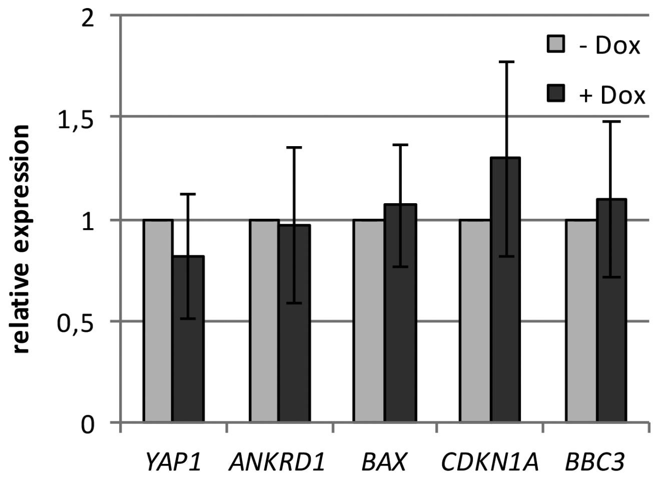

Supplementary Figure 1: Analysis of target genes in control cells. Expression of *YAP1*, *ANKRD1*, *BAX*, *CDKN1A* and *BBC3* in control TREx293 cells transfected with control plasmid *(pcDNA4TO*) after 24 h induction with 2 µg/ml Dox. Expression was analyzed by qRT-PCR and normalized to the expression of *GAPDH*.

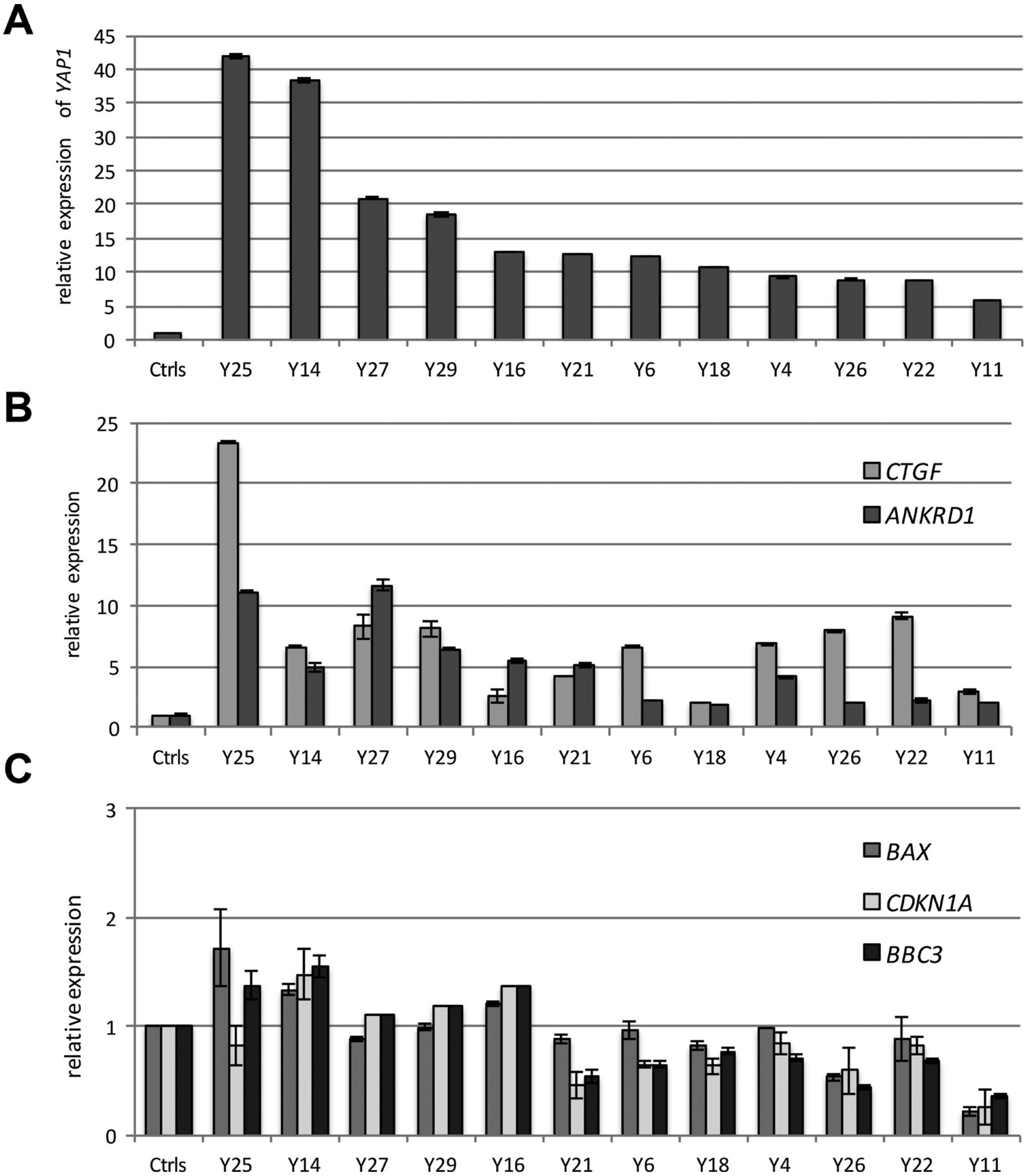

Supplementary Figure 2: Dose-dependent expression of YAP1 target genes. (**A**) Relative *YAP1* expression was analyzed by qRT-PCR and normalized to the expression of GAPDH in the indicated YAP1-TREx293 individual clones (Y#) after a 24 h induction with doxycyclin (2 µg/ml) and set in relation to uninduced cells (Ctrls = 1) (**B**) Relative expression of *CTGF* and *ANKRD1* after 24 h induction of YAP1 compared to uninduced cells (Ctrls = 1). (**C**) Relative expression of *BAX, CDKN1A* and *BBC3* after 24 h induction of YAP1 compared to uninduced cells (Ctrl = 1). All values are relative to *GAPDH* expression and to the expression without YAP1 induction (Ctrl = 1).

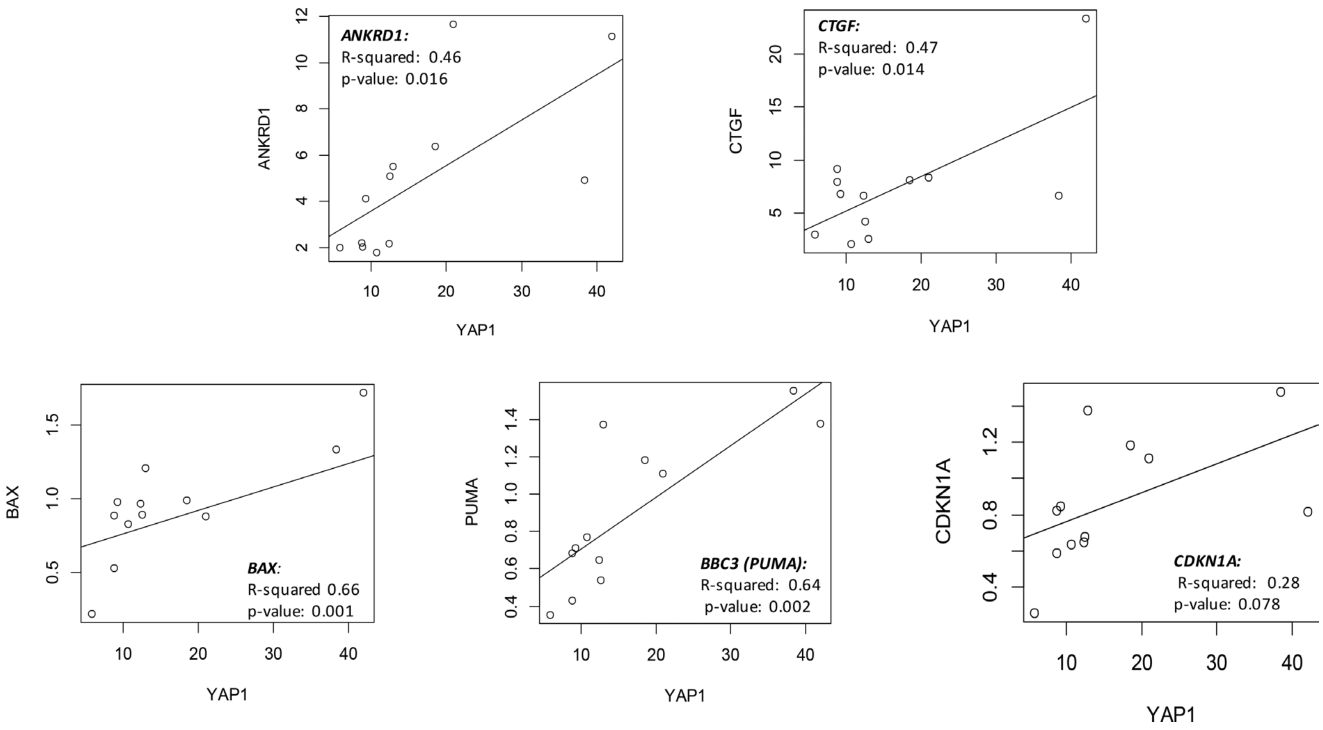

Supplementary Figure 3: Dose-dependent expression of YAP1 target genes. Correlation analysis of *YAP1* expression and *ANKRD1, CTGF, BAX, BBC3(PUMA)* and *CDKN1A* in 12 different YAP1 inducible TREx293 clones and respective *p*-values. Graphs and correlation analysis was performed by the statistical program R. For details see also supplementary Figure 2 and method section.

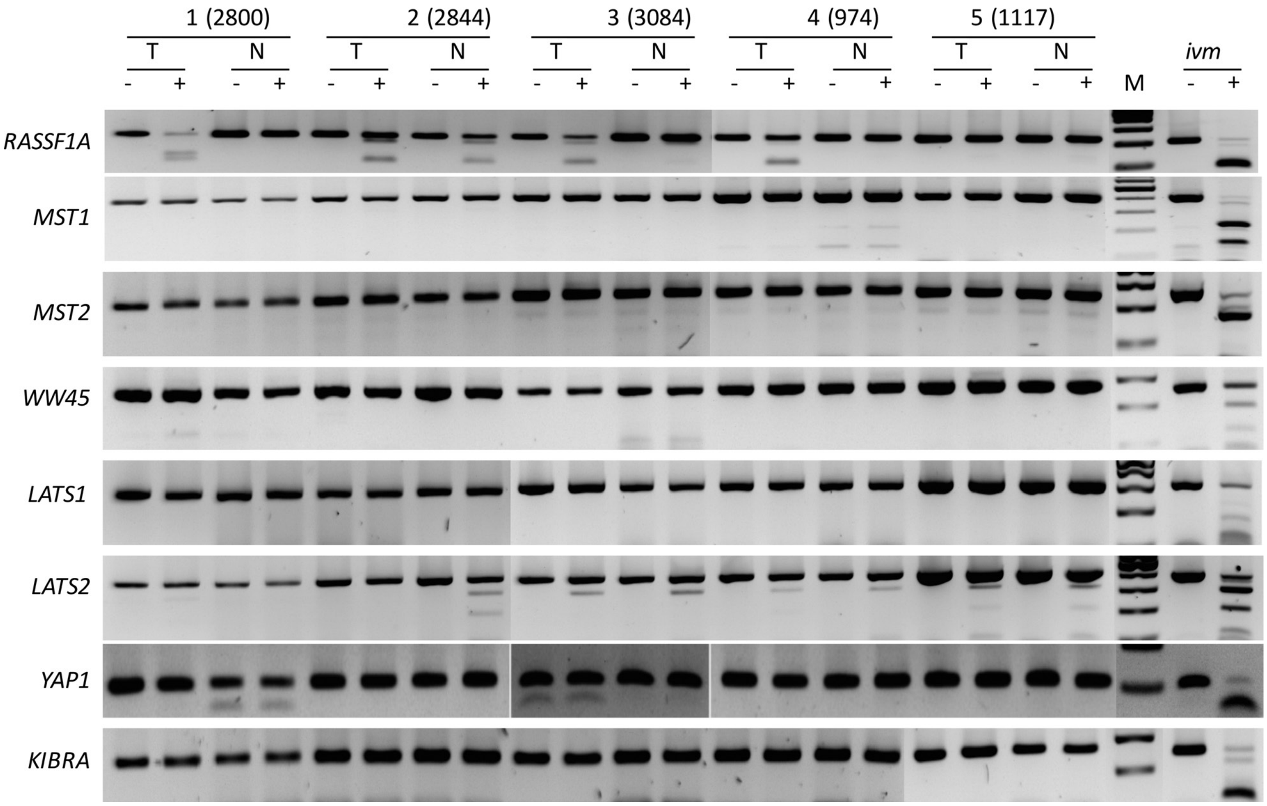

Supplementary Figure 4: Methylation analysis of the promoter region of core components of the Hippo pathway. Combined bisulfite restriction analysis of the promoter region of *RASSF1A, MST1, MST2, WW45, LATS1, LATS2, YAP1* and *KIBRA* in hepatocellular carcinomas (T) compared to matched normal samples (N). Positive control: *in vitro* methylated DNA (*ivm*). (−) mock digest; (+) PCR product digested with enzyme.

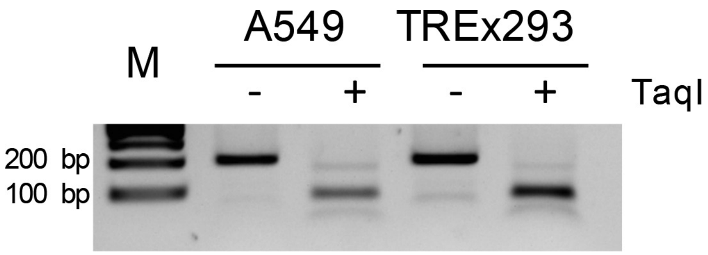

Supplementary Figure 5: Methylation of *RASSF1A* CpG island promoter in TREx293 cells. DNA from TREx293 and A549 lung cancer cells was isolated and methylation of RASSF1A was analyzed by combined bisulfite restriction analysis (CoBRA). A 205 bp fragment was amplified by semi-nested PCR utilizing bisulfite converted DNA . PCR products were subsequently TaqI (+) or mock (−) digested and resolved on a a 2% TBE agarose gel together with a 100 bp ladder.

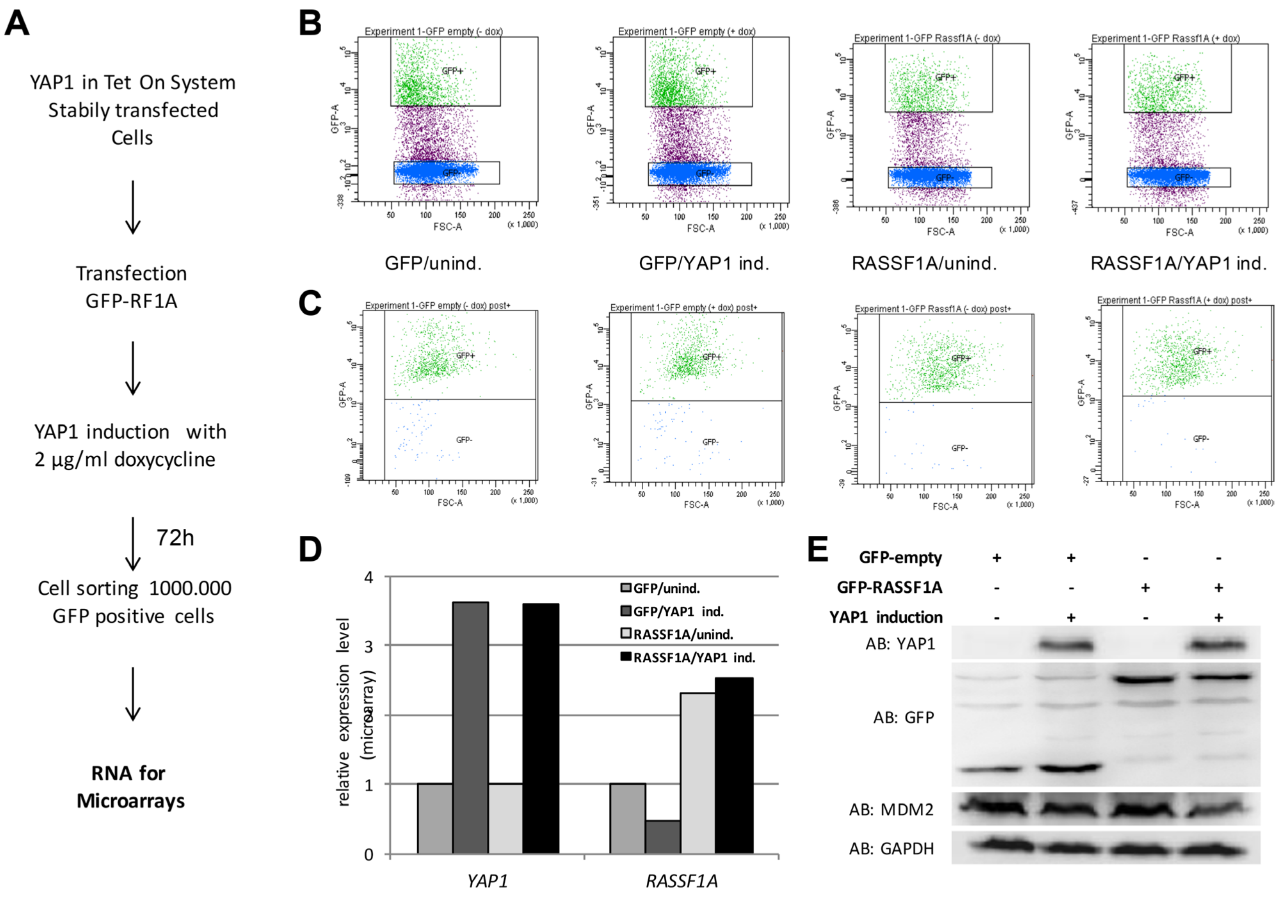

Supplementary Figure 6: Experimental design for expression analysis by microarrays. (**A**) Outline (**B**) Sorted cells in green. (**C**) Purity control from sorted cells, Green spots: sorted cells with GFP signal. (**D**) Expression of *YAP1* and *RASSF1A* in TREx293 cells was analyzed with microarrays after 72 h transfection with GFP-empty or GFP-RASSF1A and without (unind.) or with YAP1 induction (YAP1 ind).

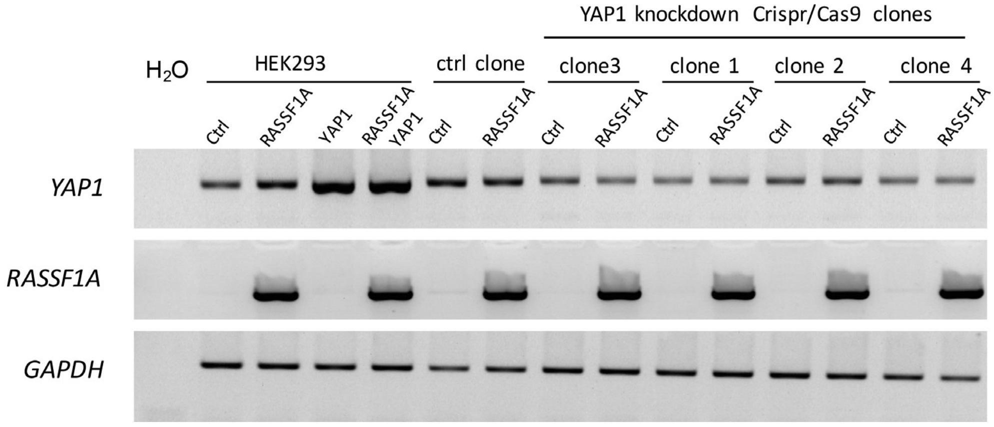

Supplementary Figure 7: Semi-quantitative RT-PCR of *RASSF1A*, *YAP1* and *GAPDH* after 72 h transfection of 4 µg plasmid or control vector (ctrl) in HEK293T cells and in YAP1 Crispr/Cas9 knockdown clones. Ctrl: GFP-empty vector; RASSF1A: GFP-RASSF1A; YAP1: Flag-YAP1. PCR products were resolved on 2% TBE agarose gels.

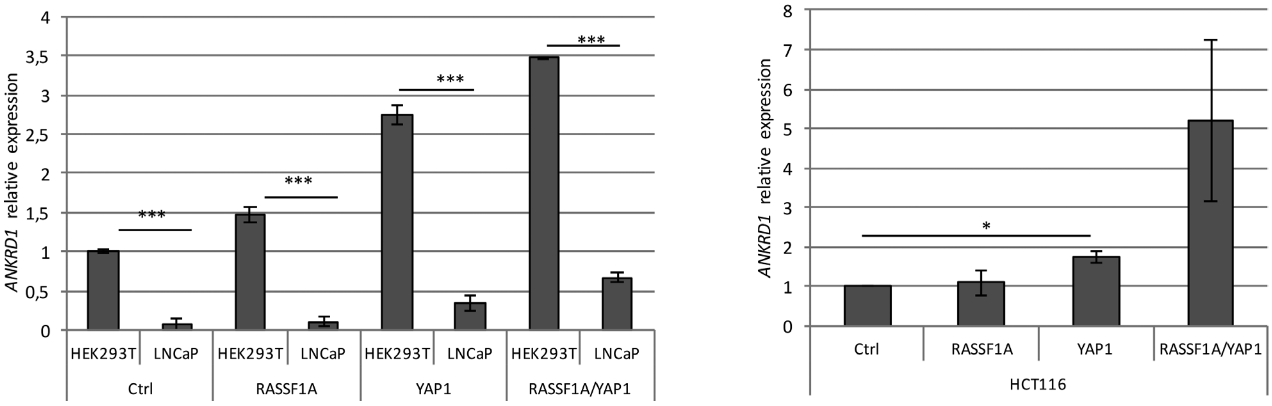

Supplementary Figure 8: RASSF1A and YAP1 induced *ANKRD1* expression. Quantitative analysis of *ANKRD1* expression level in HEK293T, LNCaP and HCT116 cells transfected with control, *YAP1* and/or *RASSF1A* after 72 h. All expression data obtained by qRT-PCR were normalized to *GAPDH* and Ctrl was set 1. *p*-values: *$p < 0.05$, ***$p < 0.001$ (*t*-test).

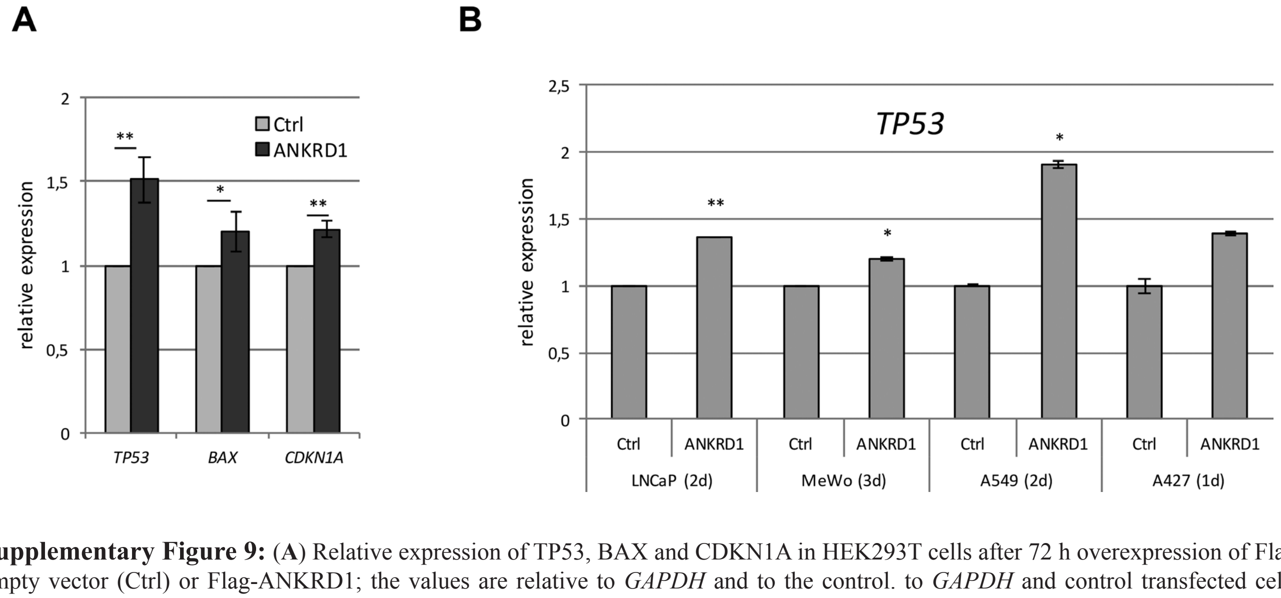

Supplementary Figure 9: (**A**) Relative expression of TP53, BAX and CDKN1A in HEK293T cells after 72 h overexpression of Flag-empty vector (Ctrl) or Flag-ANKRD1; the values are relative to *GAPDH* and to the control. to *GAPDH* and control transfected cells. (**B**) Relative expression of *TP53* after overexpression of 4 µg control plasmid or ANKRD1 in different cell lines for indicated time points (d = days). *p*-values *$p < 0.05$, **$p < 0.01$.

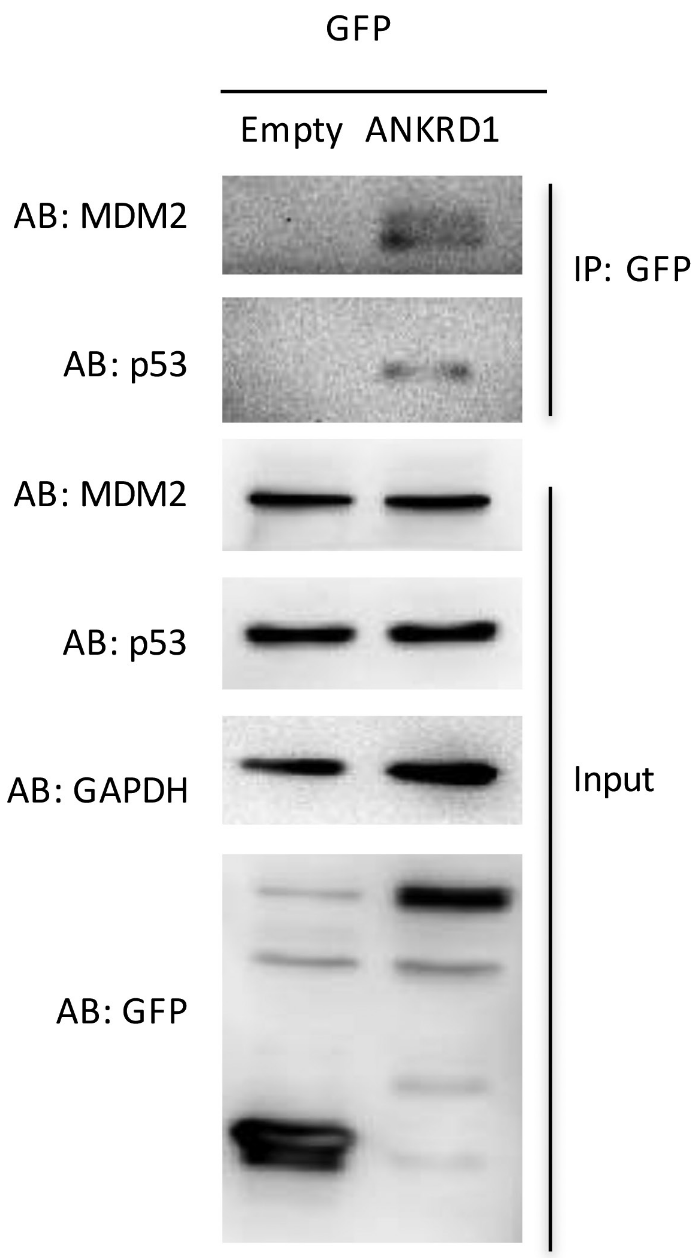

Supplementary Figure 10: ANKRD1 interacts with p53 and MDM2. Co-immunoprecipitation of MDM2 and p53 with GFP-ANKRD1. GFP-empty and GFP-ANKRD1 were transfected in HEK293 cells. After 72 h protein lysates were extracted and analyzed by Western-blot (input) with indicated antibodies (AB). Tagged ANKRD1 was precipitated with GFP-trap and the co-precipitation of MDM2 and p53 was analyzed by western blot.

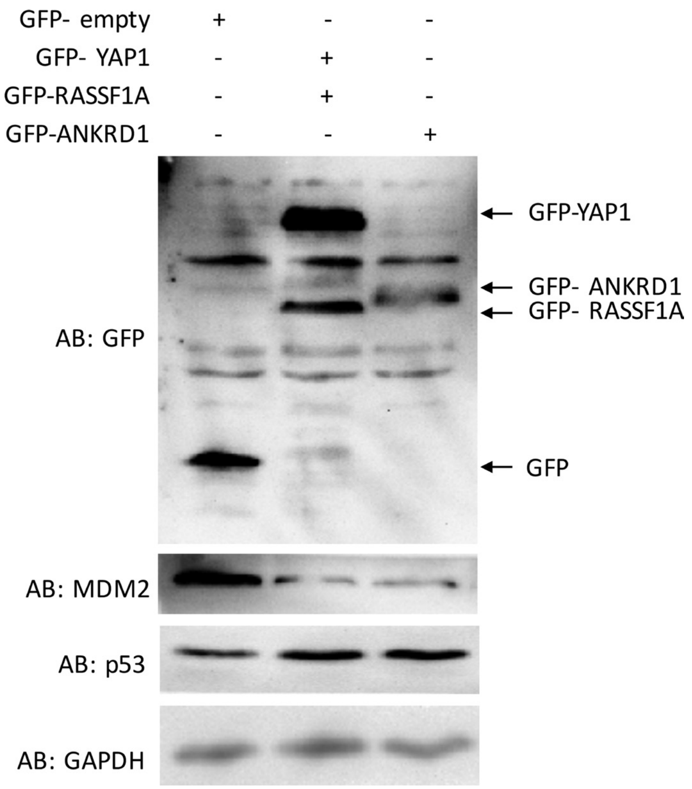

Supplementary Figure 11: ANKRD1 regulates MDM2 and p53 levels. Western blot of overexpression/co-transfection of GFP-empty, GFP-RASSF1A, GFP-YAP1, Flag-ANKRD1 in HCT116 cells. After 72 h protein lysates were extracted and analyzed by western-blot with indicated antibodies (AB).

Supplementary Table 1: primers

primer name	sequence (5′-3′)	length
**primers for bisulfite DNA**		
ANKRD1BSU1	AAGGAATTTTTGGAGTTGGTTTTGT	139 bp
ANKRD1BSL1	CACCTACCTCTAAATTAACTCCTAATAAAAA	
YAP1BSU3	TAAGGGAGTTGGAGGGAAAAAGTTTTT	98 bp
YAP1BSL3	ACRCCCCRACTCCACTAATCTAA	
MST1BSU2	GTTTGTGAAATGGGATTTAGGATTTAGG	410 bp
MST1BSL1	TCCTCTTAACCAATAACCCCTCAC	
MST2BSU1	GGGGGTGGTTAGGTTAGGTTTTGTTG	256 bp
MST2BSL2	ACTATATAACTATTAATATTTAATTCCTCCC	
UWW45B	TTYGTTGAGGATGAGTGAGGATAGT	245 bp
LWW45A	AACCAAAACCAAAACCATAATCC	
ULATS1B	AAAGGATTGAATTAATTAAGTAGGTTGGG	296 bp
LLATS1B	CCATTTTACCTTCCACTCAATATC	
ULATS2B	TGGGGGTAGATAGTAGTTAGTTAAATAGGTT	381 bp
LLATS2B	AACACTAATAACTAAAACTACTACTAACCCC	
KIBRABSU1	GGTTTTTTTTTTTTTTGGTAGGGGTAG	220 bp
KIBRABSL1	AATTTATAACTTCTACCTCCRCCAAATAC	
**primers for real time PCR**		
ANKRD1RTF1	AGCGCCCGAGATAAGTTGCT	240 bp
ANKRD1RTR1	CACCAGATCCATCGGCGTCT	
UGAP389	TGGAGAAGGCTGGGGCTCAT	176 bp
LGAP545	GACCTTGGCCAGGGGTGCTA	
UHE2ab	GGCTGGGAACCCGCGGTG	239 bp
L27111	TCCTGCAAGGAGGGTGGCTTCT	
YAP1RTFW1	TGTCTTCTCCCGGGATGTCTCAGG	215 bp
YAP1RTRW1	TGAGGGCAGGGTGCTTTGGTTG	
P53RTU1	TCAGATCCGTGGGCGTGAGCG	235 bp
P53RTL1	GGGGGTGGGAGGCTGTCAGTGG	
CTGFRTF1	CATCTTCGGTGGTACGGTGT	295 bp
CTGFRTR1	GACCAGGCAGTTGGCTCTAA	
BaxRTF	AACTGGGGCCGGGTTGTCGC	196 bp
BaxRTRew	CGCGGTGGTGGGGGTGAGG	
PUMARTFW	GCGGCGGATGGCGGACGA	188 bp
PUMARTRW	CTGACGTCCACCGGGCGGGT	
p21RTF	CCTTGTGCCTCGGTCAGGGGAG	183 bp
p21RTR	GGCCCTCGCGCTTCCAGGAC	
MDM2RTF1	ATCAGGCAGGGGAGAGTGAT	288 bp
MDM2RTR1	CCTCAACACATGACTCTCTGGA	
GADD45	CTGTGAGTGAGTGCAGAAAGC	123 bp
GADD45	CCAGCCGAGAATTCCTCCAAA	
